# The implication of LncRNA MALAT1 in promoting chemo‐resistance of laryngeal squamous cell carcinoma cells

**DOI:** 10.1002/jcla.23116

**Published:** 2019-12-14

**Authors:** Qining Jiang, Shiying Liu, Linna Hou, Yanfei Guan, Shenggang Yang, Zhengyong Luo

**Affiliations:** ^1^ Department of Radiotherapy Guizhou Cancer Hospital & The Affiliated Cancer Hospital of Guizhou Medical University Guiyang China; ^2^ Department of Otolaryngology‐Head and Neck Surgery The First People's Hospital of Yunnan Province Kunming China; ^3^ Department of Radiotherapy Yunnan Cancer Hospital & The Third Affiliated Hospital of Kunming Medical University Kunming China; ^4^ Department of Oncology Puer People's Hospital Puer China

**Keywords:** chemo‐sensitivity, epithelial‐mesenchymal transition, laryngeal squamous cell carcinoma, LncRNA MALAT1

## Abstract

**Background:**

This study was aimed to evaluate the involvement of lncRNA MALAT1 in modifying chemo‐sensitivity of laryngeal squamous cell carcinoma (LSCC) cell lines.

**Methods:**

Totally 108 pairs of tumor tissues and matched para‐tumor normal tissues were gathered from patients who were pathologically confirmed as LSCC. Meanwhile, LSCC cell lines, including TU686, TU177, AMC‐HN‐8, and LSC‐1, were purchased to evaluate their tolerance to cisplatin, 5‐fluorouracil, paclitaxel, and vincristine. Additionally, CCK‐8 assay, flow cytometry, transwell assay, and wound healing assay were implemented to assess the part of MALAT1 in modulating viability, apoptosis, invasion, and migration of LSCC cell lines.

**Results:**

MALAT1 expression was higher in LSCC tissues than in adjacent normal tissues (*P* < .05), and LSCC patients who carried highly expressed MALAT1 demonstrated poorer 5‐year survival than ones with low MALAT1 expression (*P* < .05). For another, expression of MALAT1 was also unusually elevated within TU686, TU177, AMC‐HN‐8, and LSC‐1 cell lines as relative to NHBEC cell line (*P* < .05). The TU686 cell line therein excelled in resisting the growth‐curbing effects of 5‐fluorouracil (IC50 = 20.44 μmol/L), paclitaxel (IC50 = 35.86 μg/L), and vincristine (IC50 = 0.12 μmol/L), when compared with TU177, AMC‐HN‐8, and LSC‐1 cell line (*P* < .05). Moreover, there seemed great potential for over‐expressed MALAT1 to enhance the chemo‐resistance of both TU686 and LSC‐1 cell lines (*P* < .05). Not only that, silencing of MALAT1 tended to undermine the proliferative and metastatic power of TU686 and LSC‐1 cell lines (*P* < .05).

**Conclusion:**

LncRNA MALAT1 counted in triggering tolerance of LSCC against chemo‐drugs by boosting metastasis and depressing apoptosis of tumor cells.

## 1 INTRODUCTION

Laryngeal squamous cell carcinoma (LSCC), whose prevalence was ranked as 2nd among all types of head and neck squamous cell carcinoma, accounted for up to 94 771 worldwide deaths in 2018.^1^ So far, mono‐therapies (eg surgery and definitive radiotherapy), recommended by National Comprehensive Cancer Network (NCCN), were preferred for treating T1/T2‐stage LSCC patients, whereas comprehensive projects that covered chemotherapy, radiotherapy, and surgery were prioritized for dealing with advanced‐stage LSCC patients.^2^ Despite the endeavors in attempting to overcome LSCC, the 5‐year survival of LSCC patients was still frustrating, which dropped from 66% to 63% in the past 40 years.^3^ Hence, demands for exploring mechanisms underlying LSCC development were highlighted, and quests for biomarkers that controlled LSCC progression might deliver positive consequences in LSCC diagnosis and treatment.

Given the substantial involvements in epigenetic regulation, modulation of DNA damage, and sponging of downstream miRNAs,^4^ lncRNAs were increasingly supposed as a crucial modulator of neoplastic (eg LSCC) progression. For instance, Shen et al^5^ documented that 684 lncRNAs were highly expressed, and yet 747 lncRNAs were lowly expressed within laryngeal carcinoma tissues as compared with adjacent normal tissues. It was, therefore, inferred that clarifying the expressional alteration of certain lncRNAs might conduce to identify LSCC onset and deterioration. Taking lncRNA HOTAIR as an example, appraising its serum level seemed productive in diagnosing LSCC, with a high area under the receiver operating characteristic curve (AUC) value of 72.7%.^6^ Besides, artificially suppressing HOTAIR expression was discovered to curb in vitro metastasis of LSCC cells and excessive tumor growth in LSCC mice models,^7^ which insinuated HOTAIR as a potential target for LSCC treatment. With regard to lncRNA MALAT1 concerned here, its expression in LSCC patients descended in close proportion to the rising concentration of utilized chemo‐drugs (eg cisplatin and paclitaxel) and the prolongation of treatment course.^8^ Judging by the clinical evidences, MALAT1 could have something to do with the chemo‐resistance of LSCC, although the detailed mechanisms have yet been untapped. Virtually, MALAT1 expression was observably raised in diversified neoplasms, including nasopharyngeal carcinoma,^9^ esophageal cancer,^10^ nasopharyngeal carcinoma,^11^ colorectal cancer,^12^ liver cancer,^13^ breast cancer,^14^ oral squamous cell carcinoma,^15^ and cervical cancer,^16^ which suggested that targeting MALAT1 might be fruitful in dampening neoplastic growth and improving tumor treatment. Nevertheless, there was scant information that favored the role of MALAT1 in regulating LSCC progression and also chemotherapeutic efficacy of LSCC patients.

Consequently, this investigation was arranged to expose whether MALAT1 was a promising therapeutic target for LSCC patients. In the meantime, molecular experiments were carried out to verify if modulating MALAT1 expression was capable of controlling the chemo‐tolerance of LSCC cells, which might offer a novel prospective for improving chemo‐resistance of LSCC patients.

## 2 MATERIALS AND METHODS

### 2.1 Gathering of LSCC clinical samples

From July 2013 to January 2014, totally 108 LSCC patients were recruited from Yunnan Cancer Hospital (The Third Affiliated Hospital of Kunming Medical University), and they were graded according to the criteria established by Union for International Cancer Control (UICC) in 2002. Their tumor tissues and normal laryngeal mucosa tissues (>1 cm from tumor edge) were gathered during surgery, and the extracted tissues were immediately stored in the −80°C refrigerator after being removed. It was noteworthy that the subjects have been pathologically examined as LSCC, and they did not experience radiotherapy or chemotherapy pre‐operatively. For another, the LSCC applicants were excluded from this project if: (a) their clinical and pathological information, including messages relevant to gender, age, tumor stage, treatment efficacy, and recurrence, were incomplete; (b) they were simultaneously plagued by other types of tumors; and (c) they were bothered by severe organic lesions. Moreover, this study has obtained approval from Yunnan Cancer Hospital (The Third Affiliated Hospital of Kunming Medical University) and its affiliated ethics committee. Also, informed consents were acquired from patients and their families in advance.

### 2.2 Follow‐up procedures

All the patients were followed up via telephone or outpatient review. The follow‐up period started from the date of confirmed diagnosis and lasted until demise of the patient or January 2019.

### 2.3 Cell culture

The human laryngeal cancer cell lines (ie TU686, TU177, AMC‐HN‐8, and LSC‐1) (Bena culture collection) and the normal human bronchial epithelial cells (NHBEC) were routinely cultured in RPMI1640 medium (Gibco) that consisted of 10% fetal bovine serum (FBS, Hyclone) at 37°C. Placed within an incubator of 5% CO_2_, the cells were digested with 0.25% membrane protease (Sigma) every 2‐3 days.

### 2.4 Cell transfection

Cells in the logarithmic growth phase were firstly cultivated for 12‐24 hours until they attained 60%‐80% confluence. Then, they were washed by serum‐free MEM twice before being cultured in serum‐starved conditions. Subsequently, siRNAs against MALAT1 (siRNA‐1: sense sequence: 5′‐GCAAAUGAAAGCUACCAAUTT‐3′; antisense sequence: 5′‐AUUGGUAGCUUUCAUUUGCTT‐3′; siRNA‐2: sense sequence: CGCAUUUACUAAACGCAGATT; antisense sequence: UCUGCGUUUAGUAAAUGCGTT) and pcDNA3.1‐MALAT1 (GenePharma) at a final concentration of 20 μmol/L were transfected into tumor cells, separately guided by RNAiMax siRNA transfection kit (Invitrogen) and Lipo3000 transfectamine kit (Invitrogen). After 48‐hour transfection, the cells were collected for later cellular experiments.

### 2.5 CCK‐8 assay for evaluating chemo‐sensitivity and viability of LSCC cells

Cells growing in the logarithmic phase were digested and then paved onto the 96‐well culture plates. Then, 200 μL cell suspension, which incorporated 2 × 10^3^ cells, was added into each well. The cells were cultured in 5% CO_2_ at 37°C, until they became adherent to the wall of culture plates. After 48 hours of drug treatment, cells in each well were mixed with 10 μL CCK‐8 reagent and were then incubated at 37°C for 2 hours. Ultimately, the optical density (OD) of each well was determined at the wavelength of 450 nm, and inhibition of cell proliferation was calculated based on the formula $\frac{\text{OD}450\text{control}-\text{OD}450\text{experiment}}{\text{OD}450\text{control}}\times 100\%$. A majority of procedures for detection of cell viability were identical to the above, except that medications were not delivered.

### 2.6 Colony formation assay

Cells diluted into a density of 500 per well were cultured at 37°C in an incubator (Thermo, USA) of 5% CO_2_, and the culture solution was changed every 3 days. Around 2 weeks later when macroscopic cell colonies appeared, cell culture was terminated. After rinsing the wells with PBS twice, each well was fixed by 1‐mL methanol for 30 minutes, and the cells were stained by 0.4 g/L crystal violet for 20 minutes. The number of cell colonies (>50 cells) was finally counted under the microscope.

### 2.7 Cell apoptosis assay

Cells were re‐suspended to a concentration of l × 10^6^/mL, and every 100 μL cell suspension was blended with 5‐μL annexin V‐FITC (Beckman Coulter) and 5‐μL PI (Sigma). After 15‐minute incubation in the darkness, the apoptotic percentage of 1 × 10^4^ cells was evaluated on a flow cytometry which was equipped with CellQuest software.

### 2.8 Wound healing assay for evaluating migration of LSCC cells

In the first place, we drew a vertical line in the middle of 6‐well plates utilizing the tip of a 200‐μL micro‐syringe. Then, cells were inoculated into the plates, when the time point was set as 0 hour. Forty‐eight hours later, the widths of scratches were observed under an inverted microscope and were measured utilizing Image pro plus software.

### 2.9 Transwell assay for assessing invasion of LSCC cells

Matrigel (Biosciences), diluted by MEM at a ratio of 1:2, was added to the upper Transwell chamber (Biosciences). The mixture was polymerized into the shape of gel after quiescent standing at 37°C for 30 minutes. Meanwhile, 3 × 10^4^ cells that have been configured into single‐cell suspension were also added to the upper chamber, while 500 μL serum‐containing medium was supplemented to the lower chamber. After 48 hours, cells in the upper chamber were fixed with paraformaldehyde for 3 minutes and stained by crystal violet for 5 minutes, and cells that permeated the membranes were counted under an inverted microscope.

### 2.10 Reverse transcription‐polymerase chain reaction (RT‐PCR)

Total RNAs, extracted from tissues and cell lines as per the direction of Trizol kit, were examined about their concentration and purity on a micro‐ultraviolet spectrophotometer. The qualified RNAs were reversely transcribed into cDNAs by feat of the 1st‐strand cDNA reverse transcription kit. Afterward, we relied on an RT‐PCR kit to amplify the obtained cDNAs, following the procedures of (a) 95°C for 5 minutes and (b) 40 cycles of 95°C for 10 seconds and 60°C for 45 seconds. The PCR reaction system applied (20 μL) was composed by 2‐μL cDNA, 0.4‐μL upstream primer (10 μmol/L), 0.4‐μL downstream primer (10 μmol/L), 10‐μL SYBR Green solution, and 7.2‐μL sterilized water. Besides, primers for MALAT‐1 (sense: 5′‐CAGACCACCACAGGTTTACAG‐3′, antisense: 5′‐AGACCATCCCAAAATGCTTCA‐3′) and GAPDH (sense: 5′‐TGACTTCAACAGCGACACCCA‐3′, antisense: 5′‐CACCCTGTTGCTGTAGCCAAA‐3′) were supplied by Ribobio corporation. And the expression level of MALAT‐1 was calculated by referring to 2^−ΔΔCT^ method,^17^ with GAPDH as the internal reference.

### 2.11 Western blotting

Led by the instructions of BCA protein quantification kit (Pierce), we determined the concentration of total proteins that were isolated from tissues and cell lines. Then, 20 μL total protein was allocated from each well to implement 10% sodium dodecyl sulfate‐polyacrylamide gel electrophoresis (SDS‐PAGE), as per the introductions of Bio‐Rad kit. After that, proteins on the gel were transferred onto the polyvinylidene fluoride (PVDF) membrane at a current of 80 V for 60 minutes. After blocking proteins at 37°C for 1 hour, the membrane was blended with primary antibodies (Abcam) against E‐cadherin (rabbit‐anti‐human, 1:500, Catalog No.: ab15148), N‐cadherin (rabbit‐anti‐human, 1:1000, Catalog No.: ab98952), Vimentin (mouse‐anti‐human, 1:2000, Catalog No.: ab137321), and GAPDH (rabbit‐anti‐human, 1:2500, Catalog No.: ab9485) to incubate proteins at 4°C for overnight. After 1 hour rewarming, the horseradish peroxidase (HRP)‐labeled secondary antibodies (goat anti‐mouse, 1:5000, Catalog No.: ab205719, Abcam; goat anti‐rabbit, 1:2000, Catalog No.: ab6721, Abcam) were added to further incubate proteins for another 1 hour. Developed by electrochemiluminescent kit, protein bands were photographed and detected by the alpha imaging system.

### 2.12 Statistical analyses

The data drawn from this investigation were statistically analyzed by means of SPSS 13.0 software. In particular, the measurement data expressed as mean ± standard deviation (SD) were compared by adopting one‐way analysis of variance (ANOVA) or LSD *t* test. And chi‐square test was used for contrasting enumeration data. Kaplan‐Meier and log‐rank tests were employed to estimate survival conditions of LSCC patients, and cox‐regression models were devised to figure out independent parameters that predicted survival of the LSCC patients. Notably, *P* < .05 was considered as statistically significant.

## 3 RESULTS

### 3.1 Clinical significance of MALAT1 in indicating LSCC progression

**Figure 1:**
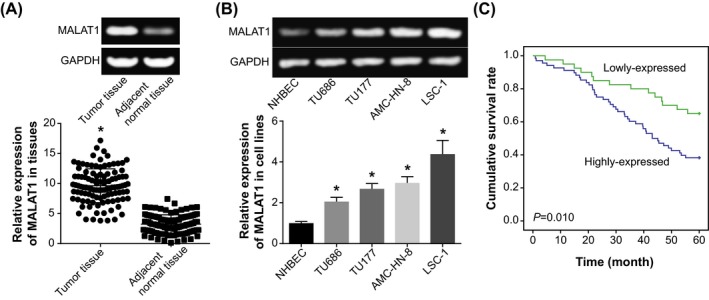
Expression of MALAT1 within laryngeal squamous cell carcinoma tissues (A) and cell lines (B), and association of MALAT1 expression with prognosis of LSCC patients (C). *P < .05 when compared with para‐carcinoma normal tissues/NHBEC cell line

RT‐PCR was applied for detecting the expression of MALAT1 in tumor tissues and para‐cancerous tissues of LSCC patients, which demonstrated that the expression of MALAT1 in LSCC tissues (ie 9.64 ± 0.82) was overtly higher than that in adjacent normal tissues (ie 3.24 ± 1.55) (*P* < .05) (Figure 1A). Identically, the expression of MALAT1 in LSCC cell lines (ie TU686, TU177, AMC‐HN‐8, and LSC‐1) also exceeded that in NHBEC cell line (*P* < .05) (Figure 1B). Moreover, the LSCC patients were further divided into the group that carried high MALAT1 expression (>median MALAT1 expression, n = 68) and the other group that embraced low MALAT1 expression (≤median MALAT1 expression, n = 40). It was suggested that high expression of MALAT1 was significantly correlative to LSCC patients who were characterized by large tumor size (>2 cm), advanced TNM grade (III‐IV), and metastatic lymph nodes (*P* < .05) (Table 1). Kaplan‐Meier curves, additionally, demonstrated that the survival condition of LSCC patients in the highly expressed MALAT1 group was poorer than that of LSCC patients in the lowly expressed MALAT1 group (*P* < .05) (Figure 1C). Besides, results of multivariate analysis elaborated that high MALAT1 expression, advanced TNM stage (III‐IV), and metastatic lymph nodes could independently mirror the poor 5‐year survival of LSCC patients (Table 2).

**Table 1:** Correlation between lncRNA MALAT1 expression and the clinical characteristic of patients with laryngeal squamous cell carcinoma

Clinical characteristics N = 108	LncRNA MALAT1 expression				χ^2^	*P* value
	Low		High			
Age						
≤50	21	52.50%	41	60.29%		
>50	19	47.50%	27	39.71%	0.626	.429
Gender						
Female	13	32.50%	15	22.06%		
Male	27	67.50%	53	77.94%	1.430	.232
Smoking history						
No	15	37.50%	20	29.41%		
Yes	25	62.50%	48	70.59%	0.752	.386
Disease site						
Glottic	31	77.50%	49	72.06%		
Supraglottic	6	15.00%	15	22.06%		
Subglottic	3	7.50%	4	5.88%	0.848	.655
Tumor size (cm)						
≤2	29	72.50%	34	50.00%		
>2	11	27.50%	34	50.00%	**5.246**	**.022**
Histologic differentiation						
Well‐moderate	32	80.00%	44	64.71%		
Poor	8	20.00%	24	35.29%	2.825	.093
TNM classification						
I‐II	33	82.50%	41	60.29%		
III‐IV	7	17.50%	27	39.71%	**5.757**	**.016**
Lymph node metastasis						
No	30	75.00%	36	52.94%		
Yes	10	25.00%	32	47.06%	**5.157**	**.023**

Bold values indicate statistically significant results with *P* value less than 0.05.

**Table 2:** Correlation between clinical characteristics and laryngeal squamous cell carcinoma patients' overall survival

Clinical features	Univariate analysis			Multivariate analysis		
	Hazard ratio	95% CI	*P* value	Hazard ratio	95% CI	*P* value
MALAT1 expression						
High vs low	5.16	2.19‐12.15	<.001	5.21	1.88‐14.46	.002
Age (y)						
≤50 vs >50	0.72	0.34‐1.55	.403	0.43	0.17‐1.12	.083
Gender						
Female vs male	1.10	0.46‐2.60	.832	1.48	0.53‐4.15	.456
Smoking history						
No vs yes	1.37	0.61‐3.09	.447	2.27	0.83‐6.18	.110
Disease site						
Glottic vs supraglottic	1.01	0.38‐2.63	.992	1.20	0.38‐3.81	.756
Glottic vs subglottic	1.47	0.31‐7.01	.626	1.28	0.20‐8.04	.791
Tumor size (cm)						
≤2 vs >2	0.90	0.42‐1.94	.795	1.67	0.64‐4.40	.298
Histologic differentiation						
Well‐moderate vs poor	0.78	0.34‐1.79	.553	1.14	0.40‐3.25	.803
TNM classification						
I‐II vs III‐IV	0.26	0.11‐0.63	.003	0.24	0.08‐0.69	.008
Lymph node metastasis						
No vs yes	0.31	0.14‐0.70	.005	0.25	0.09‐0.69	.007

### 3.2 Evaluation of LSCC cell lines' chemo‐sensitivity

**Figure 2:**
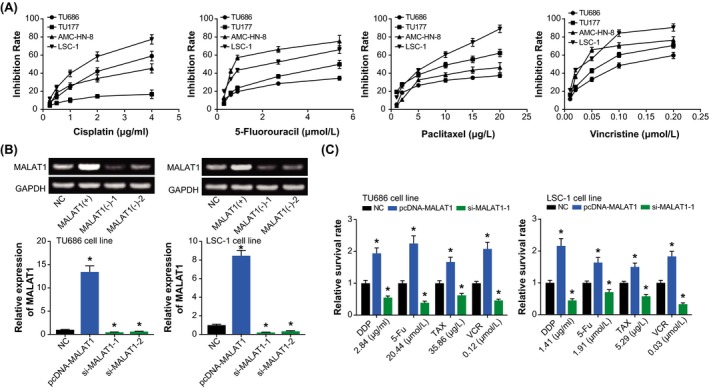
Comparison of drug sensitivity among laryngeal squamous cell carcinoma cell lines (A) and regulatory effect of the changing expression of MALAT1 (B) on chemotherapeutic tolerance in LSCC cell lines (C). *P < .05 when compared with NC

The TU686 cell line displayed the strongest tolerance to 5‐fluorouracil (IC50 = 20.44 μmol/L), paclitaxel (IC50 = 35.86 μg/L), and vincristine (IC50 = 0.12 μmol/L), when compared with TU177, AMC‐HN‐8, and LSC‐1 cell lines (*P* < .05) (Figure 2A). Furthermore, the TU177 cell line topped among all LSCC cell lines in terms of tolerance to cisplatin (IC50 = 109.08 μg/mL). As for AMC‐HN‐8 cell line, its chemo‐resistant potential seemed not outstanding, with quite moderate IC50 values for cisplatin (IC50 = 4.31 μg/mL), 5‐fluorouracil (IC50 = 1.2 μmol/L), paclitaxel (IC50 = 19.58 μg/L), and vincristine (IC50 = 0.05 μmol/L). And the LSC‐1 cell line was the least competitive in resisting against cisplatin (IC50 = 1.41 μg/mL), paclitaxel (IC50 = 5.29 μg/L), and vincristine (IC50 = 0.03 μmol/L). Since that TU686 and the LSC‐1 cell lines separately displayed the strongest and weakest tolerance to the four drugs, they were arranged for next experiments.

### 3.3 Regulation exerted by MALAT1 on the chemo‐resistance of LSCC cell lines

After transfection of pcDNA‐MALAT1, the MALAT1 expression levels in TU686 and LSC‐1 cell lines were, respectively, increased to 13.45 and 8.46 times of NC group (*P* < .05) (Figure 2B). On the other hand, si‐MALAT1‐1 inhibited MALAT1 expression more pronouncedly than si‐MALAT1‐2 (*P* < .05). When in vitro expression of MALAT1 was aggrandized, the TU686 and LSC‐1 cell lines became more resistant to cisplatin, 5‐fluorouracil, paclitaxel, and vincristine (*P* < .05) than untreated cells (Figure 2C). Conversely, si‐MALAT1‐1 further weakened the proliferative capacity of TU686 and LSC‐1 cell lines that were treated by chemo‐drugs (*P* < .05).

### 3.4 Effects of MALAT1 on growth and apoptosis of LSCC cell lines

**Figure 3:**
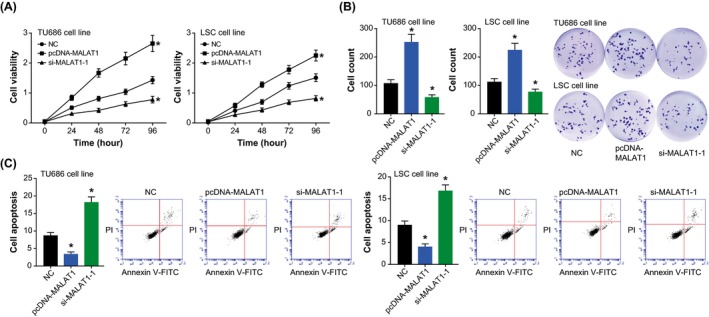
Effects of MALAT1 on viability (A), proliferation (B), and apoptosis (C) of laryngeal squamous cell carcinoma cell lines. *P < .05 when compared with NC

The viabilities of TU686 and LSC‐1 cell lines were enhanced after transfection of pcDNA‐MALAT1, when compared with NC group (*P* < .05) (Figure 3A). Opposite to over‐expressed MALAT1, under‐expression of MALAT1 reduced the viability of TU686 and LSC‐1 cells to merely 54% of the control group (*P* < .05). Furthermore, the proliferation of TU686 and LSC‐1 cell lines was intensified by transfection of pcDNA‐MALAT1 (*P* < .05) (Figure 3B), while the apoptotic trend of TU686 and LSC‐1 cells was impeded when MALAT1 was over‐expressed (*P* < .05) (Figure 3C). Additionally, the proliferative ability of TU686 and LSC‐1 cell lines, forced by si‐MALAT1‐1, was impaired significantly (*P* < .05), and their apoptotic percentage was observably elevated (*P* < .05).

### 3.5 Role of MALAT1 in regulating migration and invasion of LSCC cell lines

**Figure 4:**
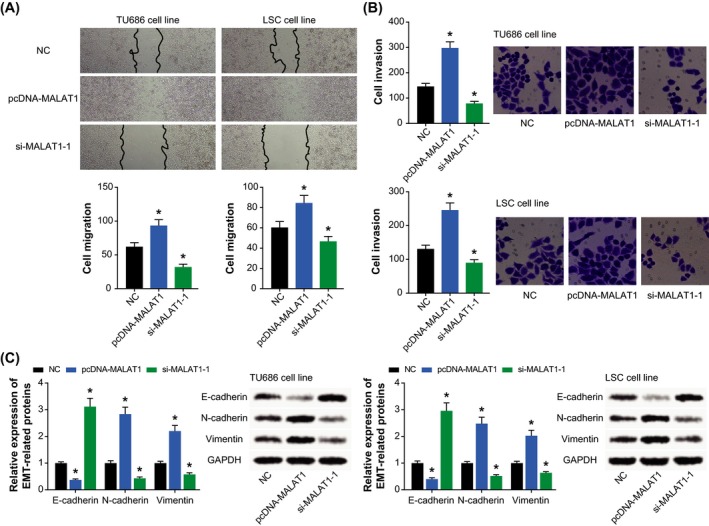
Impacts of MALAT1 on migration (A), invasion (B), and EMT‐specific protein expressions (C) of laryngeal squamous cell carcinoma cell lines. *P < .05 when compared with NC

The migratory ability of TU686 and LSC‐1 cells in the pcDNA‐MALAT1 group was distinctly improved (*P* < .05), which was exactly contrary to the si‐MALAT1‐1 group (*P* < .05) (Figure 4A). Besides, the invasive capability of TU686 and LSC‐1 cell lines was boosted by highly expressed MALAT1 (*P* < .05), yet it was weakened under the action of lowly expressed MALAT1 (*P* < .05) (Figure 4B). What's more, restraint of MALAT1 expression in TU686 and LSC cell lines triggered a rise of E‐cadherin expression and yet a drop of N‐cadherin/vimentin expression (*P* < .05) (Figure 4C). By contrast, the cell lines intentionally transfected by pcDNA‐MALAT1 were associated with lower E‐cadherin expression and higher N‐cadherin/vimentin expression than cells transfected by none (*P* < .05).

## 4 DISCUSSION

Drug resistance served as a pivotal obstacle to successful treatments of neoplasms, so elucidating pathogenesis underlying chemo‐resistance of tumor cells could provide access to improving LSCC treatments. Epithelial to mesenchymal transformation (EMT), initially proposed by Greenburg et al in 1982,^18^ referred to a phenomenon in which polarized epithelial cells were transformed into swiftly migratory mesenchymal cells. During the EMT process, expressions of epithelial markers (eg E‐cadherin) were gradually lost, while conversely, interstitial molecules (eg vimentin) were observably over‐expressed.^19^, ^20^ It was noteworthy that the invasive capability of tumor cells was significantly enhanced after induction of EMT,^21^ which associated EMT process with exacerbation of neoplasms (eg LSCC). Moreover, it might be due to its facilitating metastasis of omnifarious epithelial neoplasms^22^ that EMT functioned to urge chemo‐resistance of tumors.^23^ Taking pancreatic cancer for instance, the gemcitabine‐resistant cancer cells were discovered with typical EMT‐like phenotypes, such as abated cell adhesion and strengthened cell metastasis.^24^ Analogously, EMT‐oriented changes could also be detectable in breast cancer and ovarian cancer cells that were tolerant of paclitaxel.^25^, ^26^ More than that, tumor cells that over‐expressed EMT‐promoting transcription factors, such as Twist and Snail, became resistant to cisplatin, and knockdown of the biomarkers could sensitize cancer cells in response to the chemo‐drug.^27^, ^28^ Supported by the evidences, EMT deserved attentions in regard to investigations on chemo‐resistance of LSCC cells, and biomarkers that excelled in modulating EMT progression were promising therapeutic targets for LSCC.

Of note, multifold investigations have highlighted the involvement of dysfunctional lncRNAs in exacerbating EMT underlying carcinogenesis, and part of them were even estimated as viable treatments for tumors.^29^, ^30^ For instance, dampening HOTAIR expression was documented to changeover the resistance of hepatocellular carcinoma against cis‐platinum and doxorubicin, and simultaneously the metastatic potency of cancer cells was greatly obstructed.^31^ Concerning MALAT1 studied here, its expression was evidently fortified in esophageal cancer,^10^ cervical cancer,^16^ and nasopharyngeal carcinoma,^11^ and it also assumed EMT‐facilitating features in non–small cell lung cancer.^32^ Of note, expression of the MALAT1 was found to descend within LSCC patients who received paclitaxel‐ and cisplatin‐based treatments,^8^ insinuating the relevance of MALAT1 to drug sensitivity of LSCC. Exceeding the clinical relevance, cellular experiments performed in this study corroborated that over‐expressed MALAT1 could prompt resistance of LSCC cell lines against cisplatin, 5‐fluorouracil, paclitaxel, and vincristine (Figure 2C). Interestingly, MALAT1 was also found to reinforce the EMT potential of LSCC cells (Figure 4C), which suggested that MALAT1 could hinder chemo‐sensitivity of LSCC cells by encouraging their EMT process.

Besides EMT, flawed apoptosis of tumor cells was also one pivotal account for incremental chemo‐resistance of neoplastic cells.^33^ As illustrated by Figure 3A,B, knockout of MALAT‐1 could significantly attenuate the multiplication capacity of LSCC cell lines, and apoptosis of the LSCC cells was promoted when MALAT1 was silenced (Figure 3C). Hence, MALAT‐1 was extrapolated to improve drug‐tolerance of LSCC cells through repressing apoptosis of cancer cells. Virtually, the pro‐multiplication and anti‐apoptosis function of MALAT1 was also discoverable in other cancer cells (eg hepatocellular carcinoma and osteosarcoma),^34^, ^35^ yet whether MALAT1 acted on identical signaling pathways in distinct neoplasms entailed further evidences.

All in all, this investigation identified the likelihood that MALAT1 could contribute to incremental chemo‐resistance of LSCC by boosting proliferation and EMT of tumor cells. Nevertheless, several points could impair the reliability of conclusions drawn from this investigation. For instance, the size of clinical specimens utilized here was finite, which might fail to expose the full‐sided relevance of MALAT1 to LSCC development. Also, merely a Chinese crowd was concentrated on here, so it was risky to apply these results into people of other ethnicities. Furthermore, the impacts of MALAT1 on chemo‐sensitivity inherent in LSCC could be reflected more vividly, if mice models of LSCC were established. Last but not least, miRNAs sponged by MALAT1, such as miR‐1,^9^ should also be intensively studied, which could perfect the lncRNA‐miRNA network that accounted for LSCC deterioration.
